# Mitochondrial Dysfunction in Intensive Care Unit-Acquired Weakness and Critical Illness Myopathy: A Narrative Review

**DOI:** 10.3390/ijms24065516

**Published:** 2023-03-14

**Authors:** Felix Klawitter, Johannes Ehler, Rika Bajorat, Robert Patejdl

**Affiliations:** 1 Department of Anesthesiology, Intensive Care Medicine and Pain Therapy, Rostock University Medical Center, 18057 Rostock, Germany; 2 Department of Anesthesiology and Intensive Care Medicine, Jena University Hospital, 07747 Jena, Germany; 3 Oscar Langendorff Institute of Physiology, Rostock University Medical Center, 18057 Rostock, Germany

**Keywords:** ICUAW, critical illness myopathy, intensive care medicine, critical illness, muscle wasting, mitochondria

## Abstract

Mitochondria are key structures providing most of the energy needed to maintain homeostasis. They are the main source of adenosine triphosphate (ATP), participate in glucose, lipid and amino acid metabolism, store calcium and are integral components in various intracellular signaling cascades. However, due to their crucial role in cellular integrity, mitochondrial damage and dysregulation in the context of critical illness can severely impair organ function, leading to energetic crisis and organ failure. Skeletal muscle tissue is rich in mitochondria and, therefore, particularly vulnerable to mitochondrial dysfunction. Intensive care unit-acquired weakness (ICUAW) and critical illness myopathy (CIM) are phenomena of generalized weakness and atrophying skeletal muscle wasting, including preferential myosin breakdown in critical illness, which has also been linked to mitochondrial failure. Hence, imbalanced mitochondrial dynamics, dysregulation of the respiratory chain complexes, alterations in gene expression, disturbed signal transduction as well as impaired nutrient utilization have been proposed as underlying mechanisms. This narrative review aims to highlight the current known molecular mechanisms immanent in mitochondrial dysfunction of patients suffering from ICUAW and CIM, as well as to discuss possible implications for muscle phenotype, function and therapeutic approaches.

## 1. Introduction

Skeletal muscle weakness and muscle wasting are frequently observed phenomena in the context of critical illness [1]. The phenotypical presentation of these patients includes flaccid, symmetrical weakness of the limbs’ skeletal muscles, but respiratory muscles can be also affected [2]. Based on clinical criteria, this syndrome is termed intensive care unit-acquired weakness (ICUAW), and the diagnosis is primarily made by manual muscle strength testing using the Medical Research Council Sum Score (MRC-SS), whereby an MRC-SS < 48 defines ICUAW [3]. Currently, the clinical examination is the most frequently used diagnostic approach to detect ICUAW [4]. However, due to frequently impaired patient compliance, the clinical assessment of ICUAW may be difficult, so alternative diagnostic methods such as in vivo and in vitro biomarkers, neuromuscular ultrasound and electrodiagnostics have been investigated [5,6,7]. Their acceptance in daily clinical practice is, however, limited by the extended diagnostic effort for the ICU staff [4,8]. It must be noticed that ICUAW is an umbrella term to cover different pathophysiological conditions underlying this specific phenotype. Patients with confirmed ICUAW and documented electrophysiological neuropathy and/or myopathy can be further classified: signs of an axonal polyneuropathy define a critical illness neuropathy (CIP) and histological and/or electrophysiological evidence of a myopathy refers to a critical illness myopathy (CIM), which is considered to be the most frequent form [3]. The combined presence of CIP and CIM is, therefore, termed critical illness neuromyopathy (CINM). Clinical studies have identified several risk factors contributing to the aforementioned pathologies, of which the severity of critical illness, sepsis, multiple organ failure, prolonged immobilization and complete muscle unloading are currently considered to be the most important [9]. The pathophysiological mechanisms that link these factors to neuromuscular dysfunction have been studied experimentally in various animal models. To name a few, increased protein degradation, reduced membrane excitability, impaired autophagy and disturbed mitochondrial properties in ICUAW and CIM have been observed, suggesting that mitochondrial dysfunction contributes to the phenomenon of generalized muscle wasting [10]. Mitochondria are crucial cellular components to maintain skeletal muscle energy homeostasis in response to physiological and pathophysiological stresses [11]. Furthermore, severely impaired skeletal muscle function is a key feature of genetic mitochondrial disease, leading to a vast variety of symptoms including muscle weakness and muscle wasting [12]. Although the flaccid palsy seen in ICUAW itself is not merely the result of mitochondrial dysfunction, it is very likely to contribute to the patients’ debilitating general weakness, increased fatigability and reduced exercise capacity.

Therefore, this narrative review aims to highlight the current known molecular mechanisms immanent in mitochondrial dysfunction of patients suffering from critical illness-induced muscle wasting, as well as to discuss possible implications for muscle phenotype, function and therapeutic approaches.

## 2. Mitochondrial Structure and Function in Healthy Skeletal Muscle

Mitochondria can be found in the majority of eukaryotic cells, as they are essential organelles to maintain cellular metabolism and energy supply. In skeletal muscle fibers, their subcellular localization can be either subsarcolemmal (SSM) or intermyofibrillar (IMF) [13]. According to the primary source of energy supply, skeletal muscle fibers can be distinguished into different types with varying mitochondrial content [14]. Slowly contracting type I muscle fibers primarily rely on oxidative metabolism, and therefore contain many mitochondria. Fast-contracting type II muscle fibers can be further differentiated into type IIA fibers, using both oxidative and glycolytic metabolism for energy generation, and type IIB fibers, which are mainly dependent on glycolysis. In humans, the type IIB-specific myosin heavy chain is not expressed, and the respective fiber type should, consequently, be named “IIx” [15]. In both type II fibers, mitochondria are relatively sparse compared to type I fibers. Mitochondrial dysfunction can be assumed to have pronounced effects on type I fibers, consistent with atrophy of these fibers in inactivity and critical illness [16,17]. On the other hand, a dominant atrophy of type II fibers due to a lower mitochondrial volume has been proposed to occur in aging human muscles, but elaborate morphological studies have questioned this view in recent decades [18].

Irrespective of differences in the reductions in mitochondrial content and shifts between different fiber types in skeletal muscle, it is clear that the increased ATP hydrolysis during contractions needs to be balanced by ATP synthesis at some point: Whereas this task is reached at the local level in type I fibers, it is delegated to the liver by type II fibers. Thus, whenever considering the energy demands of the muscles, it is mandatory to take account of the mitochondrial function of hepatocytes and—for the effort of transporting oxygen and metabolites—cardiomyocytes. In both organs, severe alterations occur during sepsis [19,20].

**Figure 1:**
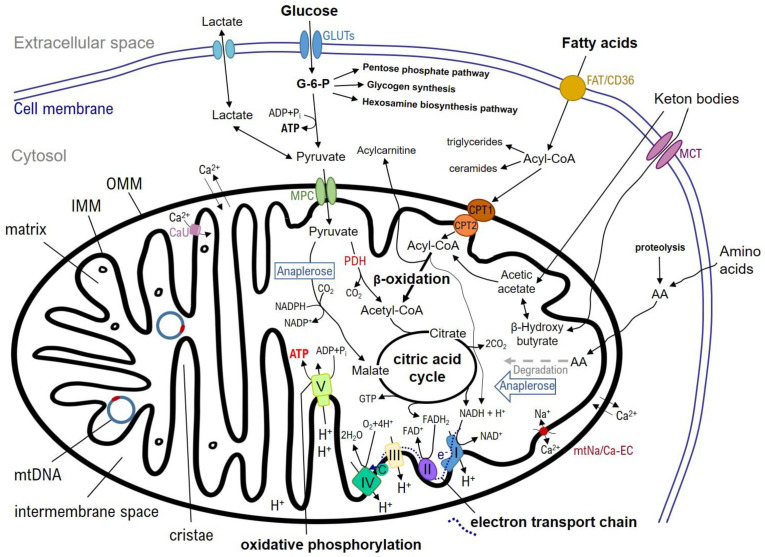
Schematic representation of the main metabolic pathways in mitochondria. The most important functions of the mitochondria include the citrate cycle, the β−oxidation of fatty acids and the mitochondrial respiratory chain with electron transport chain and oxidative phosphorylation. AA: amino acids, Acyl−CoA: acyl−coenzyme A, ADP: adenosine diphosphate, ATP: adenosine triphosphate, C: Cytochrome C, CaU: mitochondrial calcium uniporter, CTP: carnitine palmitoyl transferase (1 and 2), FAD: flavin adenine dinucleotide, FADH_2_: flavin adenine dinucleotide hydroquinone, FAT/CD36: fatty acid translocator/cluster of differentiation 36, GLUTs: glucose transporters, GTP: guanosine triphosphate, G−6−P: glucose−6−phosphate, H^+^: hydron, IMM: inner mitochondrial membrane, MCT: monocarboxylate transporter, MPC: mitochondrial pyruvat carrier, mtDNA: mitochondrial desoxyribonucleic acid, mtNa/Ca-EC: mitochondrial sodium−calcium exchanger, NAD^+^: nicotineamid adenine dinucleotide, NADH: nicotineamide adenine dinucleotide hydroquinone, NADP^+^: nicotineamide adenine dinucleotide phosphate, NADPH: nicotineamide adenine dinucleotide phosphate hydroquinone, OMM: outer mitochondrial membrane, PDH: pyruvate dehydrogenase, I: NADH dehydrogenase, II: succinate dehydrogenase, III: cytochrome c reductase, IV: cytochrome c oxidase, V: ATP synthase.

Likely due to their ancient evolutionary origin from bacterial predecessors, mitochondria contain their own genome consisting of a circular double-stranded deoxyribonucleic acid (DNA) with 37 genes encoding for mitochondrial proteins, especially subunits of the respiratory chain complex and several transfer ribonucleic acids (RNA) [21]. They are double membrane structures only a few micrometers in diameter, housing a great variety of enzyme complexes and transporter proteins integrating them into multiple molecular pathways (Figure 1). The outer mitochondrial membrane (OMM) is a double-layered phospholipid membrane separating the mitochondrion from the cell cytosol. It contains various proteins which are essential key structures in mitochondrial dynamics, as well as several carriers to import substrates for mitochondrial metabolism. Separated by the intermembrane space, mitochondria contain the inner mitochondrial membrane (IMM), which covers the mitochondrial matrix. The IMM is invaginated multiple times to form the cristae, where the enzymes of the electron transport chain (ETC) are located. Mitochondria are frequently referred as the “power plants” of the cell, because they are the main source of adenosine triphosphate (ATP) generation for cellular energy supply [21]. Therefore, in the first step, Acetyl-CoA is generated from either pyruvate by oxidative phosphorylation (OXPHOS) executed by the enzyme pyruvate dehydrogenase, or by β-oxidation of free fatty acids (FFAs). Afterwards, Acetyl-CoA enters the tricarboxylic acid (TCA) cycle to create the energy-rich substrates nicotine amide dinucleotide (NADH) and flavin adenine dinucleotide (FADH2), which donate electrons via the enzymes of the ETC (complex I—NADH dehydrogenase, complex II—succinate dehydrogenase, complex III—cytochrome c reductase, complex IV—cytochrome c oxidase) to oxygen as a final electron acceptor [11]. This electron transfer is coupled with a hydrogen ion flux from the mitochondrial matrix into the intermembrane space. Thus, an electrochemical gradient is gradually build up to finally fuel the ATP synthase (complex V), which generates ATP from adenosine diphosphate (ADP).

## 3. Mitochondrial Dynamics in Healthy Skeletal Muscle

**Figure 2:**
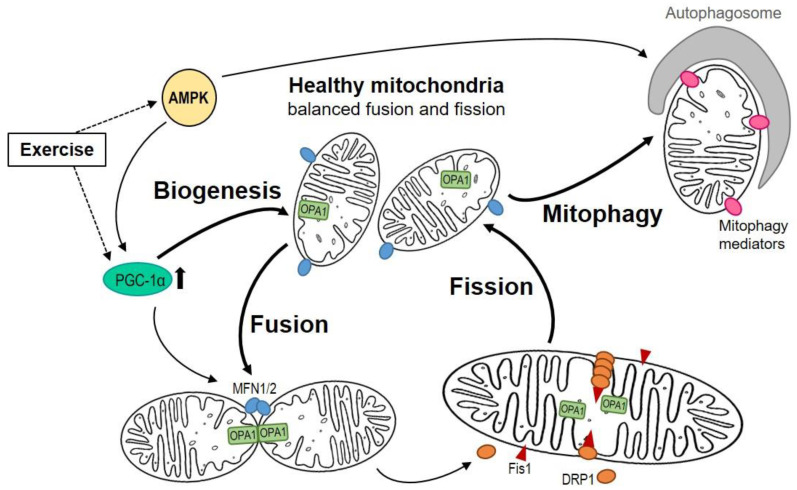
Relationships between mitochondrial biogenesis, fusion, fission and degradation in healthy mitochondria. Muscle mitochondria underlie extensive turnover and remodeling in response to a diverse panel of physiological inputs. Among the many activators of PGC1α, AMPK seems to be the most crucial for regulating mitochondrial metabolism and biogenesis. PGC1α activates mitochondrial biogenesis and regulates mitochondrial dynamics by controlling the expression of MFN1, MNF2 and DRP1. Mitochondrial fusion is mediated by MFN1/2 for the fusion of the outer membrane and OPA1 for the inner membranes. Mitochondrial fission is controlled by DRP1 and Fis1. DRP1 is predominantly located in the cytosol and is targeted to the surface of mitochondria, where it binds to Fis1. This complex acts as a potential scission site for fission. Damaged or dysfunctional mitochondria are eliminated by mitophagy facilitated by different mediators. AMPK: AMP—activated kinase, DRP1: dynamin-related protein 1, ETC: electron transport chain, Fis1: fission protein 1, MFN1/2: mitofusin1/2, OPA1: optic atrophy 1, PGC1α: peroxisome proliferator—activated receptor gamma coactivator alpha.

Mitochondria are highly dynamic organelles, undergoing permanent changes to their structural organization by fusion, fission and degradation in response to various stimuli to maintain and adapt their physiological function (Figure 2) [22]. This is part of a quality control program where new mitochondria are created in a process called biogenesis, and damaged or dysfunctional organelles are removed by mitophagy [23]. In brief, mitochondrial biogenesis starts with the replication of mitochondrial (mt) DNA. This is a complex process orchestrated by numerous proteins and signaling cascades, in which peroxisome proliferator-activated receptor gamma coactivator alpha (PGC1α) is considered to be the key regulator [24]. In the first step, PGC1α is activated by phosphorylation through the enzyme AMP-activated protein kinase (AMPK). In a downstream cascade, PGC1α activates the nuclear respiratory factors (NRF) 1/2, which in turn promote the expression of the mitochondrial transcription factor A (TFAM) and the mitochondrial transcription factors B1 (TFBM1) and B2 (TFBM2). They are translocated into the mitochondrion, initiating mtDNA replication and mtRNA transcription by promoting attachment of DNA and RNA polymerases [22,25]. Mitochondrial fusion is a process whereby two or more organelles are combined to form networks to fit energetic requirements or compensate for isolated enzyme dysfunction [26]. Therefore, fusion of the OMM is regulated by the GTPases mitofusin 1 (MFN1) and mitofusin 2 (MFN2), whereas fusion of the IMM is mediated by the protein optic atrophy 1 (OPA1) [22,27]. In skeletal muscle fibers, mitochondrial organization and fusion dynamics seem to differ between muscle fiber types. Whereas fast-twitch muscle fibers are described to have a block-like compartmentalization of their mitochondria, slow-twitch fibers seem to present elongated and interconnected networks between distinct sarcomeres [28]. In contrast, mitochondrial fission is the separation of fused organelles into distinct entities. Thus, the dynamin-related protein 1 (DRP1) binds to the OMM by interacting with different receptor proteins (e.g., mitochondrial fission 1-Fis1, mitochondrial fission factor-MFF, mitochondrial dynamics protein of 49kDa-MID49, MID59) and initiates constriction and separation of the OMM and IMM [22,29].

## 4. Assessment of Mitochondrial Function

As mitochondrial integrity is crucial for cellular bioenergetics and mitochondrial dysfunction has been shown to participate in a great spectrum of disease entities, various approaches have been taken in the detection and monitoring of mitochondrial function. Thus, it can be roughly divided into in vitro and in vivo diagnostic methods, targeting different functional units within the mitochondria [30]. In an attempt to link current practical trends in modern intensive care medicine and recent advances in the assessment of mitochondrial function at the molecular and metabolic levels, we would like to discuss three promising in vivo diagnostic approaches for mitochondrial function.

Magnetic resonance spectroscopy (MRS) is a specialized technique of common MR-imaging, which quantifies electromagnetic signals emitted by atoms or molecules within a certain region of interest [31]. With MRS, metabolic processes can be assessed in vivo using certain tracers, depending on the chemical composition of participating reactants. In regard to mitochondrial metabolism, ^13^C-MRS can be used to determine utilization of carbohydrates and lipids via the TCA cycle and ^31^P-MRS for evaluation of mitochondrial ATP metabolism via the assessment of phosphocreatine turnover [31]. MRS has also been used to evaluate ATP metabolism in primary motor neuron disease [32] and animal models of sepsis [33], revealing impaired mitochondrial bioenergetics. However, according to expert opinion, MRS can only reliably quantify ATP turnover in exercising muscles, but seems inaccurate in resting muscle due to imbalances between ATP flux and actual ATP production [34]. Furthermore, from practical reasons due to highly specialized and bulky equipment, the application seems primarily suitable for research purposes, but inappropriate in daily intensive care practice.

Near-infrared spectroscopy (NIRS) is an in vivo optical method that utilizes the ability of heme groups to absorb infrared light by different amounts depending on their oxygenation status for the quantification of the overall oxygen saturation in the local tissue volume below [30]. This principle can be used to indirectly estimate mitochondrial oxidative capacity via assessment of muscle oxygen consumption. Therefore, the NIRS sensor is applied above a region of interest with the muscle below. Following muscular exercise, short serial occlusion maneuvers lead to intermittent secessions of blood flow, whereas changes in oxygen saturation only depend on muscle oxygen consumption, which is directly proportional to mitochondrial respiratory capacity [35]. In the case of reduced mitochondrial oxidative capacity, re-oxygenation after exercise is assumed to be faster [36]. This method provides several advantages, including its non-invasive and easy application, and has been successfully used to detect disturbed mitochondrial function in aging, myopathies and muscular dystrophies [35,37]. However, in critically ill patients, several limitations might impair its diagnostic accuracy. According to Adami et al. the rate constant of muscle oxygen consumption depends on tissue oxygen concentration, so the oxidative capacity can only be reliably estimated when oxygen is abundantly available [38]. In conditions of critical illness, such as heart failure, shock and sepsis, microcirculation and, therefore, tissue oxygenation can already be severely impaired, theoretically hampering NIRS measurements [39]. Additionally, due to prevailing bioenergetic failure in critical illness, it could be impossible to raise mitochondrial enzyme activity by exercising to a maximum that is comparable to healthy conditions, theoretically overestimating the oxidative capacity assessed by NIRS. Furthermore, uncertainties regarding the actual penetration depth of the infrared light in case of edema or a thickened subcutaneous fat layer may bias NIRS measurements.

The protoporphyrin IX-triplet state lifetime technique (PpIX-TSLT) is another optical approach to assess mitochondrial function in vivo. The aim of this non-invasive method is to estimate the mitochondrial oxygen tension, which is assumed to be a surrogate parameter for the balance between mitochondrial oxygen supply and demand [40]. Therefore, 5-aminolevulinic acid can be administered to the skin, which leads to the formation and accumulation of protoporphyrin IX in the underlying tissue mitochondria. When stimulated, the emission of red light from the protoporphyrin IX can be detected; the lifetime of the fluorescence is inversely related to mitochondrial oxygen tension [40]. This innovative approach has been already evaluated in conditions of critical illness using animal models of sepsis, with promising results [41,42]. Furthermore, the cellular oxygen metabolism monitor (COMET), a novel device which incorporates the principle of the PpIX-TSLT, has recently been evaluated in critically ill patients and seems feasible for measuring mitochondrial oxygen tension at the bedside [43]. However, as this technique quantifies mitochondrial oxygen tension locally in a small area of tissue, estimates for mitochondrial function in an entire organ or body might not be accurate. Furthermore, due to the limited penetration depth of light pulses, detected signals may rather reflect mitochondrial function from superficial tissues than deeper skeletal muscles. Therefore, the diagnostic and prognostic value of this new in vivo approach has yet to be determined for mitochondrial function in skeletal muscles, especially in critically ill patients.

## 5. Structural and Functional Impairment of Mitochondria following Immobilization

Different attempts have been made to evaluate changes in mitochondrial structure and function following pure immobilization and unloading of skeletal muscles. Frequently used methods include cast immobilization, hindlimb suspension, denervation and neural cord dissection [44,45,46,47]. Therefore, it appears that SSM is more vulnerable than IMF to mechanical unloading in limb skeletal muscles, leading to severely impaired mitochondrial membrane integrity [47,48,49,50]. In contrast, following mechanical ventilation, severe morphological alterations, including fragmentation, shrinking and loss of branched architecture, were observed mainly in diaphragmatic IMF, but to a much lesser degree in SS [51]. This could be explained by the fact that passive stretching of muscle fibers is already present under mechanical ventilation. Aside from morphological alterations, overall mitochondrial content and mtDNA have been found to be significantly reduced following immobilization in skeletal muscles of the limbs [52,53,54]. Furthermore, mitochondrial turnover seems to be impaired. Essential factors promoting mitochondrial fusion (including PGC1α, MFN1, MFN2 and TFAM) are downregulated in the majority of studies following immobilization in limb skeletal muscles [44,45,51,53,55]. These findings might support an impaired mitochondrial fusion process. However, data regarding changes in pro-fission factors (e.g., DRP1) are heterogeneous, showing reduced [53], increased [51,56] and no changes [57], which might be explained be different methods of muscle unloading and applied durations of immobilization.

At the functional level, the majority of investigations seem to demonstrate an overall decrease in mitochondrial ETC enzyme protein concentration, enzyme activity, respiratory capacity and ATP production [44,46,47,48,49,53,56,57,58,59,60]. Furthermore, it appears that besides reduced mitochondrial energy production, dysregulation of the OXPHOS system and uncoupling of the ETC lead to increased formation of ROS. This has been hypothesized by numerous studies demonstrating increased H_2_O_2_ concentrations and overshooting activation or depletion of antioxidant defense systems [44,47,48,49,56,58]. These results are underpinned by studies observing attenuated mitochondrial protein damage and restoration of protein expression by antioxidant treatment after immobilization [61].

Mitophagy also seems to be impaired after denervation, possibly promoting accumulation of dysfunctional mitochondria and further increasing oxidative stress [62]. However, these results could not be observed using hindlimb suspension even after four weeks of immobilization, suggesting a more severe impact of nerval disruption on muscle bioenergetics [53].

## 6. Structural and Functional Impairments of Mitochondria in ICUAW and CIM

### 6.1. Animal Models of CIM and Mitochondrial Dysfunction

The use of animal experiments is common practice in critical care research to mimic and study distinct features of critical illness, as it provides several advantages such as high reproducibility, control of environmental parameters and the absence of interfering chronic disease or therapeutic interventions [63]. In an attempt to deepen the understanding of the basic pathophysiological mechanisms leading to muscle and mitochondrial dysfunction under conditions of critical illness, different animal models have been developed in recent decades [10]. However, the translation of results from animal studies to human properties is not possible without restrictions, as some biological and methodical aspects need to be discussed. Variations in the (a) study settings, (b) investigated animal species and (c) skeletal muscles themselves need to be compared to clinical studies on critically ill patients with ICUAW and CIM.

In an attempt to replicate hallmark features of muscle weakness and wasting in critical illness, several different methods, including mechanical denervation, with or without steroids [64,65,66,67]; induction of sepsis [68,69,70,71,72]; and sedation and mechanical ventilation with or without paralytics [65,73] have been investigated either individually or in combination [69,74,75]. Although the majority of studies were able to mimic certain pathophysiological aspects, it has been found that the combined effect and prolonged application of some methods was best suited to replicate predominant myosin loss, decreased compound muscle action potentials and normal nerve conduction velocities, similar to patients with CIM [69,74,75]. In this context, porcine animal models of critical illness revealed marked downregulations in mitochondrial gene expression controlling the PGC1-family, the ETC enzyme complexes and several metabolic pathways [73,76], matching at least in part with the results of patients with CIM [77,78]. Although not covering all methodical aspects of a fully grown ICU animal model, studies investigating mitochondrial function by pure immobilization [52,53,54], denervation [47,49,59] or sepsis [79,80] also show deficits in mitochondrial structure and function comparable to observations in critically ill patients, including mitochondrial swelling, decreased mitochondrial content and downregulation of PGC1α mRNA expression as well as several mitochondrial metabolic pathways [81,82,83].

The majority of experimental studies have used mice [68,69], rats [47,84] and pigs [65,71,74,75,76] in their animal models to investigate the impact of different conditions of muscle unloading and neuromuscular impairment on muscle integrity and function. Human physiology shares many similarities with these mammalians, of which porcine physiology is considered to be closest to humans. However, it must be kept in mind that certain aspects, e.g., the skeletal muscle transcriptome and metabolome, can significantly differ between species. Mitochondrial oxidative capacity, mRNA expression of respiratory chain complexes and metabolic coupling vary between mice, rats and humans [85]. Furthermore, despite the preservation of many physiological mechanisms among mammalians, the onset of age-related muscle wasting and subsequent changes in muscle phenotype as well as muscle transcriptome can vary between species and even within certain strains of one single species [86]. These findings imply that the biological characteristics of an animal model should match the requirements of the scientific hypothesis, and that the researcher should know about the advantages and disadvantages of a certain model organism.

Comparing animal models and human studies of ICUAW and CIM also demands discussion of individual skeletal muscle properties. In clinical studies of CIM and mitochondrial dysfunction, the majority of biopsies have been taken from the vastus lateralis muscle [1,78,82,83,87,88,89,90]. This contrasts with many ICU animal models, in which a great variety of limb skeletal muscles, including the soleus muscle [16,64,65,69,75,84], the gastrocnemius muscle [47,64,75], the extensor digitorum longus muscle [65,69,70,75,91], plantaris muscles [16], the biceps femoris muscle [73,76] and the tibialis anterior muscle [64,65,68,75], have been assessed. This is important to recognize, as human and animal skeletal muscles do not necessarily share the same anatomical characteristics. The skeletal muscles of human limbs show a relatively uniform fiber composition, with equal distributions of type I and type II fibers [92]. In contrast, skeletal muscles in rodents and pigs may contain predominantly type I or type II fibers [16,93,94,95]. Whereas the human vastus lateralis contains about 40% type I and 60% type II fibers [96], the porcine as well as the rat vastus lateralis consists of approx. 80% type II fibers [16,93,94,95].

Both biomechanical and metabolic properties may vary between human and animal skeletal muscles. Early works demonstrated the different activity levels of ETC complex II between different animal species within the same muscle fiber types [97,98]. Furthermore, it has been shown that in humans, the enzymatic activity of ETC complex II within one muscle fiber type may vary between different skeletal muscles of the limbs [98]. These findings have been corroborated by Murgia et al., who demonstrated that the metabolic setup (mitochondrial content and mitochondrial enzyme activities) can significantly differ between individual muscle fiber types [99]. In line with this, the mitochondrial content and PGC1α levels within muscle fibers from mice and rats seem not to correspond to humans [100]. In conclusion, ICU animal models offer several advantages to study certain aspects of CIM and contributing mitochondrial dysfunction, but inherent biological and methodical issues limit the translation to human physiology, especially in critically ill patients.

### 6.2. Mitochondrial Content and Morphology Is Altered in Critical Illness

**Figure 3:**
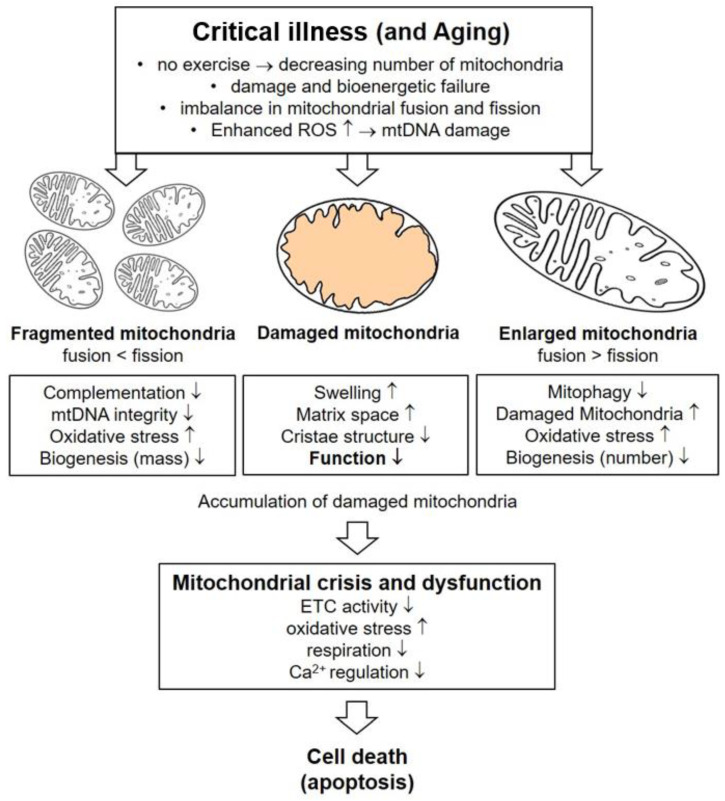
Impact of critical illness on mitochondrial dynamics in skeletal muscle cells. Interference with the homeostatic regulation of mitochondrial fusion and fission pathways, resulting in abnormal mitochondrial morphology. The presence of fragmented mitochondria due to a decrease in fusion and/or an increase in fission may compromise mtDNA integrity, mitochondrial structural and functional complementarity and mitochondrial biogenesis. Each one can lead to mitochondrial dysfunction. Conversely, the formation of enlarged mitochondria as a result of decreased fission and/or increased fusion events may decrease mitochondrial turnover by impairing mitophagy and biogenesis. This leads to the accumulation of damaged mitochondria in the cells. Critical diseases can cause bioenergetic failure, increased formation of ROS and imbalances in homeostatic regulation which damage mitochondria, leading to swelling, increases in matrix space, decreases in cristae structure and decreases in functionality. Accumulation of dysfunctional mitochondria in the cells finally results in cell death. ETC: electron transport chain, mtDNA: mitochondrial desoxyribonucleic acid, ROS: reactive oxygen species.

Bioenergetic failure related to critical illness seems to be accompanied by an overall reduction in mitochondrial content and function (Figure 3). In an animal model of severe burn injury (with full-thickness burns, 60% of the total body surface area), a 36% decrease in the number of mitochondria was observed [101]. Similar results have been demonstrated in septic mice; a loss of about 30% was seen in limb and respiratory skeletal muscles [102]. This results are in line with data from critically ill patients with sepsis and MOF, in whom overall mitochondrial degradation was evident [13].

Furthermore, not only a diminished number of mitochondria, but also ultrastructural changes in the remaining organelles point to severe alterations in mitochondrial integrity during critical illness. Mitochondrial swelling, a fragmented or nearly absent cristae structure, matrix space enlargement and vacuolization are the most frequent morphological pathologies that have been reported in animal [79,80,103] and human [13,104] skeletal muscle biopsies associated with critical illness-induced muscle wasting. In accordance with the rapid development of muscle weakness and the degradation of muscle mass due to critical illness, the decrease in mitochondrial content can be observed within several days after ICU admission [83]. Besides the inflammatory and metabolic challenges, critical illness is accompanied by physical inactivity, a factor which, by itself, is strongly associated with mitochondrial alterations [105].

### 6.3. Impaired Biogenesis, Mitophagy and Mitochondrial Regeneration

Dysregulation of mitochondrial dynamics has already been linked to critical illness-induced organ dysfunction. In sepsis, dysfunction of multiple regulating proteins contributes to impaired mitochondrial biogenesis and recovery. Inhibition of mitochondrial fusion by suppression of MFN2 and extensive mitochondrial fission by upregulation of DRP1 may promote multiple organ failure [106]. In relation to skeletal muscle homeostasis, experimental defects in MFN1 and MFN2 have been shown to directly promote muscle wasting [107]. Furthermore, recent evidence has suggested hampered translocation of TFAM, as an initiator of mtDNA replication, to be associated with altered mitochondrial turnover [108]. PGC1α, the key regulator in mitochondrial biogenesis, also seems to be affected during critical illness. In an animal model of sepsis, expression of PGC1α mRNA was significantly reduced in limb and respiratory skeletal muscles, and limb muscles seem to be more vulnerable [80]. Critically ill patients often experience bedrest and muscle disuse due to mechanical ventilation and sedation. Prolonged immobilization steadily decreases PGC1α, NRF1/2 and TFAM protein expression, as well as mitochondrial content, in skeletal muscles [109]. Although the availability of studies investigating mitochondrial biogenesis in patients with ICUAW and CIM is limited, this is in line with data from 33 patients at risk for ICUAW who presented marked reductions in skeletal muscle PGC1α mRNA expression, suggesting impairments in mitochondrial biogenesis that were already profound during the early phase of critical illness [90]. The relevance of PGC1α, TFAM and NRF1/2 as regulators for mitochondrial recovery and restoration of skeletal muscle function has been underlined in early investigations, demonstrating an upregulation of these biogenesis promotors in critical illness survivors [82,110].

Mitophagy is a specialized form of autophagy which allows the removal of dysfunctional or damaged mitochondria and related protein complexes to maintain cellular homeostasis. This process is facilitated by the parkin E3 ligase, an enzyme promoting degradation of dysfunctional mitochondria [111]. Impairments to this cellular quality control mechanism can severely disrupt mitochondrial integrity, leading to failure of skeletal muscle contractile function [112]. Therefore, mitophagy seems to be affected to different extents in limb and respiratory skeletal muscles [80]. This is underlined by results showing that improvements in autophagy by artificial parkin overexpression resulted in preserved mitochondrial quality control and prevented myofiber atrophy [112].

### 6.4. Dysregulation of the ETC Complex

Alterations to the ETC enzyme complexes in skeletal muscle have frequently been observed in connection to critical illness and muscle wasting [11]. Studies evaluating rodent animal models of sepsis (induced either through the injection of lipopolysaccharide (LPS) or by performing cecal puncture and ligation) have reported a functional decrease in various enzyme complexes. From a methodological point of view, it should be mentioned that the measurement of enzyme activities does not necessarily reflect the functional status of mitochondria. An overall reduction in organelles can also contribute to reductions in enzyme activities. However, to distinguish functional alterations of enzyme complexes from pure changes in overall mitochondrial count, signal intensities have to be compared to a reference enzyme within the same cellular compartment. For assessment of the ETC enzyme complex, the activity of the citrate synthase, which is located in the mitochondrial matrix, can be used as a reference [88,113]. In this context, a recent study reported a profound downregulation of the enzyme activities of complex II, III and IV in mechanically ventilated and LPS-treated mice diaphragm muscle strips [114]. However, the results were expressed as activity per gram protein, making conclusions in regard to the function status of the ETC enzymes difficult. In another investigation, Oliveira and coworkers reported a marked downregulation of all ETC enzyme complex mRNAs, as well as a reduced protein expression of the complexes III and IV in the diaphragms of septic mice [102]. Skeletal muscles of not only the respiratory system, but also of the limbs, experience significant alterations in their OXPHOS systems. In an experimental study on mice, functional deficits of ETC enzymes in limb skeletal muscles persisted for weeks after the onset of sepsis, and were accompanied by a significant reduction of complex I driven electron transport capacity and activity of the ETC complexes II and IV [79]. Therefore, it has been suggested that, along with other mitochondrial impairments, a reduction in the ETC complex activity seems to contribute to persistent muscle weakness, independent of inflammatory status and accompanied muscle atrophy [79]. However, these results must be interpreted with caution, because no data regarding the level of physical activity during the observational period have been reported. In this context, overall severe organ damage (e.g., brain and heart) rather than mitochondrial failure might have contributed to physical inactivity and disability, leading to muscle weakness.

Although it seems obvious that reduced ETC enzyme activity is related to a reduction in muscle strength and function, studies investigating the relationship between integrity of the OXPHOS system, muscle strength and function in critically ill patients with ICUAW are sparse and partly inconsistent. Some studies examined the function and integrity of the OXPHOS system in biopsies from limb skeletal muscle in critically ill patients. Early investigations by Brealey et al. demonstrated significantly reduced ATP concentrations, but no differences in skeletal muscle citrate synthase-normalized ETC enzyme activities between sepsis survivors and non-survivors [89].

Duceau and coworkers used proteome and metabolome analysis to demonstrate a marked downregulation in OXPHOS gene expression in septic patients [81]. However, a depletion of mitochondrial protein content suggests that an overall reduction in mitochondrial count rather than specific reductions in ETC enzyme activities were reported in other studies [13,82]. As the patients’ muscle strength was not assessed in either study, the clinical significance remains elusive. Jiroutková and coworkers assessed the ETC enzyme complex function in a cohort of ICUAW patients [88]. It was found that citrate synthase-normalized enzyme activities of complex I and IV were not different from healthy controls. Surprisingly, an increase in enzyme activity of complex II and III was observed, which was explained by a compensatory switch in mitochondrial nutrient utilization. Unfortunately, the study by Jiroutková et al. did not compare critically ill patients with and without ICUAW with each other. Furthermore, no correlation between mitochondrial dysfunction and muscle strength was established. Based on these first observations, they reproduced their results in a following study, again with muscle biopsies from ICUAW patients, but without direct correlation to muscle strength and function [87]. The findings of Jiroutková and coworkers are in line with a recent study investigating ETC enzyme activities in stroke patients with ICUAW [113]. Although the activity of ETC complex I was increased compared to postoperative controls, no comparisons between critically ill patients with and without ICUAW were made. Taken together, despite some evidence from animal models suggesting alterations in the ETC complex activity in the context of critical illness-induced skeletal muscle dysfunction, data from critically ill patients with ICUAW and CIM are sparse and at least partly divergent.

### 6.5. Increased Oxidative Stress

Besides its function as the most important source of ATP, the mitochondrial respiratory chain also contributes to the production of reactive oxygen species (ROS), which are thought to participate physiologically in cell signaling [115]. However, damage to the respiratory chain can induce ROS overproduction, leading to oxidative stress, disruption of cellular organelles and, finally, apoptosis. Due to the ubiquitous presence of mitochondria, neuromuscular dysfunction may not solely result from ROS-associated damage within muscle fibers. Instead, ROS-mediated alterations at the level of the neuromuscular synapses, motor nerves and the central nervous system are known to contribute to weakness in a number of neuromuscular diseases [116,117,118].

At the level of the neuromuscular junction (NMJ) of motor nerve terminals, a ROS-mediated inhibition of transmitter release has been described [119]. Whereas the function of postsynaptic structures seems to remain intact, presynaptic disruption of the NMJ was thought to lead to distal motor neuron degeneration and consecutive muscle denervation by ROS [120]. Not only an overproduction of ROS, but also the downregulation of major antioxidant defense systems such as the superoxide dismutase (SOD) 1 and 2, which are located within mitochondria, might impair the neuromuscular function, especially at the NMJ and connected nerve terminals. In various studies using SOD-knockout animal models, significant damage to the NMJ and adjacent nerve terminals has been demonstrated [121,122,123,124]. Therefore, motor neurons seem to be more affected than sensory nerves [124]. However, in the condition of increased oxidative stress not exclusively mitochondrial, but also ROS from other cellular compartments might contribute to neuromuscular failure [125].

In the skeletal muscles of patients suffering from critical illnesses, the ROS-associated damage may persist even after sepsis has been overcome [79]. Furthermore, not only an overproduction of ROS, but also a downregulation of antioxidant systems seems to be at hand in critical illness [81,115]. In skeletal muscles, the combined effect of muscle unloading and systemic inflammation could further augment oxidative stress and downregulate antioxidant defense mechanisms such as SOD 2 [102]. Besides the direct downregulation of gene expression, the inability to translocate nuclear transcription factors that are essential for mitochondrial protein synthesis could also be hypothesized as a potential mechanism in antioxidant response failure [108]. A downregulation of antioxidant systems, such as the thioredoxin/peroxiredoxin, glutaredoxin 5 and glutathione systems, has already been described in patients with ICUAW [81,89]. The compensatory upregulation of genes and proteins regulating oxidative homeostasis, such as PGC1α, TFAM and SOD2, are seen in sepsis survivors in response to increased oxidative stress and ROS production, and might be a possible explanation for resilience and recovery in critical illness [13,82,126]. Among other important functions controlling mitochondrial homeostasis, PGC1α has been suggested to be a master regulator in this antioxidative response [127]. Furthermore, activation of the Janus kinase (JAK)/signal transducer and activator of transcription proteins (STAT) pathway may also contribute to increased oxidative stress in skeletal muscle mitochondria [115]. In this context, recent evidence has suggested interleukin-mediated JAK/STAT activation as a potential mechanism for muscle wasting in sepsis [128].

### 6.6. Mitochondrial Calcium Homeostasis and Dysregulation

Calcium ions are indivisibly linked to proper skeletal muscle structure and function by fueling the contractile apparatus, acting as intracellular signaling molecules and impacting muscle fiber plasticity [129]. In recent decades, mitochondria have been recognized as crucial components shaping calcium dynamics in myocytes [130]. Therefore, mitochondrial contribution to calcium homeostasis obviously differs between certain types of muscle fibers [131]. In contrast to mainly glycolytic type II fibers, mitochondria actively participate in controlling intracellular calcium concentrations in slow-contracting oxidative type I fibers [132,133]. Mitochondria can transiently store high amounts of calcium and, therefore, might act as calcium-buffering organelles [134]. Furthermore, mitochondrial function and intracellular calcium control seem to impact the excitation–contraction coupling in skeletal muscles. A study by Eisner and coworkers demonstrated that the inhibition of MFN causes alterations in myocyte calcium dynamics, impairing active muscle contraction [135]. Furthermore, PGC1α also takes part in regulating mitochondrial calcium homeostasis by preventing the age-related downregulation of genes, including those encoding for the mitochondrial calcium uniporter (MCU) [136]. Therefore, besides various other proteins, the MCU seems to be responsible for the majority of mitochondrial calcium influx [137,138,139,140]. The crucial role of transient mitochondrial calcium uptake through the MCU has recently been underlined by Gherardi et al., who demonstrated that a depletion of the MCU impairs skeletal muscle force generation and glycolysis and induces a slow-to-fast fiber switch [141]. Furthermore, increased mitochondrial FFA oxidation as well as intensified hepatic glycogenolysis and ketone body production emerged in this study, reflecting widespread alterations in mitochondrial nutrient utilization capabilities. In aged individuals, a decrease in MCU activity was linked to increased mitochondrial ROS production, and restoration of MCU function enhanced antioxidant defense [142]. The involvement of mitochondria in shaping myocyte calcium signaling via interactions with the sarcoplasmatic reticulum is, overall, a finely tuned system of machinery, whereby transient fluctuations in mitochondrial calcium concentrations occur physiologically [143]. However, dysfunctional mitochondria are unable to limit excitation-induced calcium transients, leading to impaired control of overall cytoplasmatic calcium concentrations. Subsequently, states of chronically increased intramitochondrial calcium may occur, promoting enzyme dysfunction and organelle damage and ultimately inducing cell death by apoptosis [144,145]. One mechanism which likely contributes to this fatal cascade includes altered opening kinetics of the mitochondrial permeability transition pore (mPTP) in response to impaired mitochondrial calcium handling [146]. The mPTP is located within the IMM and can translocate pro-apoptotic factors such as the Bcl-2-associated X protein (Bax), triggering the release of cytochrome c and activation of caspase 9 and 3 and inducing apoptosis [147,148,149]. Investigations by Csukly et al. have already demonstrated that following muscle denervation, myocyte calcium concentrations increased significantly, leading to calcium overload, and facilitated mPTP opening [150]. These findings were complemented by Karam et al., who proposed the absence of calcium transients as a trigger for mitochondrial dysfunction and demonstrated that the reoccurrence of calcium transients is essential for restoring the opening kinetics of the mPTP [151]. Following muscle denervation, mitochondrial calcium transients cease, causing increased mPTP opening and ROS production. Another mechanism possibly contributing to impaired calcium transients was proposed by Friedrich et al., who showed inhibition of sarcoplasmatic reticulum calcium release by IL1α, promoting muscle weakness in CIM [152]. In contrast, transient openings of the mPTP have been proposed to protect mitochondria from calcium overload by releasing calcium into the cytoplasm [153]. Disturbances to this finely orchestrated regulation of mitochondrial calcium homeostasis certainly appear under conditions of critical illness. In animal models, the induction of sepsis by either LPS or CPL was associated with a reduction in mitochondrial calcium transients in cardiomyocytes [154], as well as increased mPTP opening, causing myocardial damage [155]. Furthermore, sepsis-induced mitochondrial calcium overload contributes to impairments in mitochondrial respiratory chain activity, possibly promoting contractile dysfunction and muscle weakness [70,156]. The first evidence for impaired skeletal muscle calcium homeostasis in critically ill patients with CIM was reported by Friedrich and coworkers [157]. In this context, the complete mechanical silencing frequently observed in patients with ICUAW and CIM impairs overall myocyte calcium dynamics by inhibition of sarcolemmal calcium release [158]. However, studies from critically ill patients directly investigating mitochondrial calcium dynamics are still lacking, leaving most of the experimental findings presented herein uncertain.

### 6.7. Relations to Mitochondrial Gene Expression and MicroRNAs

Various conditions of critical illness have been linked to mtDNA damage and profound alterations in gene expression encoding for key regulators in mitochondrial function [90,159,160]. Muscle biopsies from critically ill patients revealed an overall decrease in mtDNA content and a downregulation of many genes orchestrating mitochondrial function [90,161]. This was also recently confirmed in patients with CIM [77]. Especially genes for mitochondrial fusion and replication, such as PGC1α, MFN2 and OPA1, seem to be affected in skeletal muscles [102,114]. Therefore, PGC1α mRNA expression is already significantly downregulated in skeletal muscles within the first days after ICU admission, indicating impairments in mitochondrial biogenesis and turnover in the early phase of critical illness [78,90]. In a recent animal model of septic mice, it was shown that IL-6 may play a crucial role in the suppression of PGC1α mRNA expression, suggesting a direct involvement of cytokines in sepsis-mediated muscle wasting [127]. Another essential regulator of mitochondrial replication dynamics is TFAM. As a core transcription factor, it directly interacts with mtDNA, covering and stabilizing entire DNA regions and controlling RNA polymerase interaction, as well as, therefore, gene expression [162]. Similar to PGC1α, a marked decrease in TFAM protein levels has been observed in sepsis-induced diaphragm damage, which might be the result of a failure of translocation of precursor TFAM into mitochondria for final initialization rather than nuclear gene suppression [108,114]. Reductions in or complete abolishment of TFAM have been associated with marked decreases in mtDNA, leading to profound skeletal muscle wasting [163]. The important role of TFAM in preservation of muscle mass is underlined by the fact that TFAM overexpression has been shown to protect from hindlimb suspension-induced muscle wasting [164]. In contrast, in survivors of critical illness, an upregulation in the mRNA of PGC1α, NRF1 and TFAM could be indicative of ongoing repairing processes or, on the other hand, could serve as a mechanism of resilience [82]. This may be strengthened by recent findings showing an upregulation of peroxisome proliferator-activated receptor gamma (PPARγ) signaling in sepsis, through which PGC1α could express its anti-inflammatory function [81].

Another mechanism proposed to disrupt mitochondrial function in critical illness might be mediated through microRNAs (miRNA). These non-coding RNA molecules are assumed to regulate protein expression by translational repression, a post-transcriptional mechanism in the modulation of gene expression [165]. Their involvement has been extensively described for many different conditions of muscle wasting, including muscular dystrophies [166], cancer cachexia [167] and aging [168]. Furthermore, they seem to play a crucial role in critical illness-induced muscle wasting. Therefore, miRNA can be either factors thought to protect from or to promote muscle wasting through their regulation of different key proteins of mitochondrial biogenesis, such as PGC1α and NRF1 [169]. It is noteworthy that possible differences in molecular regulation patterns between different species can produce contradicting results. In an animal model of sepsis, miRNA 181a seems to promote muscle wasting through the direct suppression of mitochondrial function and reduction in mtDNA content [104]. In contrast, miRNA 181a has been shown to protect from muscle wasting by counteracting insulin-like growth factor (IGF)-15 in critically ill patients [170]. However, a widespread network of differently expressed miRNAs may play a potential role not only in critical illness-induced muscle wasting, but also in muscle regeneration and recovery by targeting mitochondrial regulators. In patients with ICUAW, miRNA 542-3p/5p was supposed to induce muscle wasting through a depression in mitochondrial 12S/16S ribosomal RNA [171]. Downregulation of miRNA-424-3p/5p in quadriceps muscle biopsies, for example, has been shown to correlate with alterations in muscle mass, muscle strength and overall physical function in the acute phase of critical illness. Patients recovering from muscle wasting and regenerating muscle mass showed miRNA expression patterns distinct from those of patients who were unable to regain muscle mass [172].

### 6.8. Metabolic Changes and Mitochondrial Dysfunction

Mitochondria are essential components for generating energy to fuel cellular function by integrating the final steps in the metabolism of glucose and lipids. Acetyl-CoA, the main substrate for the TCA cycle, is generated by either pyruvate from glycolysis or β-oxidation of fatty acids. Disturbances in the metabolism of glucose or fatty acids have frequently been observed in critical illness, and are inextricably linked to altered mitochondrial function and skeletal muscle homeostasis [173].

Adequate glucose utilization is vital for preserving mitochondrial function in skeletal muscles, and is mediated via the action of insulin-activating subsequent signaling pathways [174]. Regularly, insulin binds to its receptor and induces autophosphorylation, which allows for the recruitment of insulin receptor substrates (IRS) 1 and 2. IRS are able to activate phosphoinositide 3-kinase (PI3K), which results in the phosphorylation of plasma membrane lipids generating various phospholipids, which are able to bind and activate phosphoinositide-dependent kinase 1 (PDK1). PDK1, in turn, can activate the serine/threonine kinase AKT, which is considered a checkpoint enzyme integrated in many different intracellular signaling cascades, including glucose transporter type 4 (GLUT4) and the forkhead box (FOXO) transcription factors [174]. Furthermore, intact insulin signaling not only preserves adequate glucose uptake to fuel the TCA, but also directly maintains mitochondrial function by inducing mitochondrial protein and DNA synthesis, stimulating ATP production and promoting FFA oxidation [175,176].

The term “insulin resistance” describes the failure of insulin to induce subsequent metabolic pathways, resulting in impaired cellular glucose uptake, inadequate inhibition of gluconeogenesis and downregulation of anabolic processes. It is frequently associated with hyperinsulinemia, hyperglycemia and increased concentrations of FFAs. In reference to mitochondrial integrity, states of chronic hyperglycemia and insulin resistance have been directly linked to skeletal muscle mitochondrial dysfunction, including reduced mitochondrial content, reductions in OXPHOS activity and OXPHOS gene expression, dysregulation of mitochondrial biogenesis through suppression of PGC1α and ROS overproduction in diabetes mellitus [177,178]. In this context, is should be mentioned that mitochondrial dysfunction itself can lead to insulin resistance [179]. Not only in chronic disease, but also in acute conditions of critical illness, insulin resistance has been linked to impaired mitochondrial biogenesis [180]. The phenomenon of insulin resistance was highlighted as a metabolic hallmark feature in critical illness myopathy and ICUAW [90,181,182]. Furthermore, overall gastrointestinal function may be impaired especially in critically ill patients with neuromuscular alterations [183]. In muscle biopsies from critical ill patients with CIM, partial impairment of the insulin receptor-mediated signaling cascade was demonstrated [78]. Despite preserved AKT activation, insulin was unable to promote glucose utilization, suggesting a subsequent failure of insulin signaling downstream of AKT. As mentioned earlier, AKT can phosphorylate and thereby inhibit members of the FOXO transcription factor family. Subsequent disinhibition of FOXO transcription factors has been demonstrated in animal models of sepsis, theoretically providing a possible linkage to mitochondrial dysfunction [91,184]. Increased FOXO activity indirectly impairs mitochondrial function by induction of the heme oxygenase 1 (HMOX1), which cleaves heme that is, therefore, unavailable in the ETC enzymes [185]. Furthermore, FOXO directly suppresses PGC1α, a key regulator of mitochondrial biogenesis. Therefore, impairments of AKT-mediated FOXO suppression in critical illness may directly hamper mitochondrial function and promote muscle wasting. Furthermore, insulin-independent translocation of GLUT4 via activated AMPK was shown to be diminished in CIM patients [78,182]. The AMPK is another enzyme interlinking glucose metabolism, mitochondrial function and skeletal muscle integrity. Depending on the prevailing situations, AMPK has been shown to be a kind of “double edged” sword, on one hand promoting protein degradation, mitophagy and muscle wasting, and on the other hand protecting from muscle wasting and promoting muscle regeneration by maintaining mitochondrial integrity. Therefore, exercise-induced activation of AMPK stimulates mitochondrial biogenesis through activation of PGC1α and NRF [177]. The protective effects of AMPK on muscle mass have recently been underlined in sepsis [84]. However, whether disturbed glucose metabolism is a major cause or only a bystander effect in the pathogenesis of CIM remains controversial [186].

The metabolism of lipids provides another essential pathway for the generation of ATP that converges in mitochondria. Glucose and lipid metabolism are inextricably interlinked with each other, preserving mitochondrial energetic integrity and muscle health. Therefore, in conditions of critical illness, alterations in glucose metabolism are frequently accompanied by disturbances in lipid homeostasis, aggravating skeletal muscle wasting. In states of increased energy demands, lipids provide a quickly available fuel source for the organism. When glucose utilization is impaired and glycogen stores are depleted, skeletal muscle derives energy from the metabolism of FFAs and ketone bodies [187]. Lipolysis is increased in sepsis involving pro-lipolytic hormones, as well as enzymes such as perilipin 1 and hormone-sensitive lipase for the breakdown of triacylglycerol, which is the storage form of lipids in adipocytes [173]. During this process, FFAs are released to meet the increased energy demands. However, utilization of FFAs through β-oxidation is impaired in the early course of sepsis, possibly by the downregulation of PPARγ signaling, a key regulator in gene expression for mitochondrial lipid utilization. The imbalance between high fuel influx and impaired utilization capacity can lead to uncontrolled accumulation of intracellular lipids, leading to a cytopathic effect called lipotoxicity [173]. Thus, the intramuscular accumulation of lipids can induce muscle damage and may impair muscle regeneration [188]. Increased intracellular concentrations of FFAs have been shown to hamper mitochondrial ATP generation, and lipotoxicity can directly impair mitochondrial function [189]. The proposed mechanisms of lipotoxicity are ROS overproduction and OXPHOS uncoupling [190]. Furthermore, the accumulation of FFAs and lipid intermediates can lead to insulin resistance in skeletal muscles [191]. The activation of stress-induced kinases such as protein kinase C, IkB kinase and c-Jun N-terminal kinase, which phosphorylate and, therefore, inactivate IRS, has been proposed as a possible mechanism in impaired insulin signaling [177]. In line with these findings, β-oxidation enzyme concentrations are reduced in the skeletal muscles of patients with ICUAW within the first days of critical illness [90]. In the skeletal muscle of from septic patients, mitochondrial fatty acid degradation is reduced within the first days [81]. Increased levels of circulating acyl carnitine derivatives within the first postoperative week in intensive care patients with muscle wasting indicate impaired utilization of lipids in β-oxidation rather than hampered mitochondrial import [192]. In the condition of prolonged critical illness, remaining mitochondria seem to adapt to the increased lipid supply after the first week. A metabolic switch in mitochondrial energy utilization in sepsis from glucose to FFA has recently been described [114,193]. Increased activity of ETC complex II, which receives electrons mainly from β-oxidation, suggests augmented utilization of FFA for ATP production [88]. Furthermore, fatty acid oxidation capacity was demonstrated to be significantly increased in the presence of FFAs in patients with ICUAW [87]. However, in contrast to this hypothesis, recent studies demonstrated no changes or lower activities of ETC complex II concentrations and activities in skeletal muscles during prolonged critical illness in ICUAW patients and septic animals [79,113].

## 7. Dynamics of Mitochondrial Alterations and Therapeutic Implications

Alterations in the skeletal muscles’ mitochondrial structure and function can be observed within the first few days after ICU admission. Mitochondrial swelling, decreased mitochondrial content, a significant decline in muscle ATP and phosphocreatine concentrations as well as pathologically reduced activities of the ETC enzymes I and IV have been described [82,89,90]. In line with this, severe impairments in mitochondrial metabolic pathways including OXPHOS, TCA, ketone utilization and β-oxidation show up within the first three days [81]. These findings are accompanied by early derangements at the gene level. Sepsis survivors showed marked increases in PGC1α mRNA and NRF1 mRNA expression compared to non-survivors, implying insufficient activation of mitochondrial biogenesis contributing to adverse outcomes in critical illness [89]. Within the first two weeks, mitochondrial dysfunction may be aggravated in prolonged critical illness and have insufficient regenerative capacities, as a further decline in mitochondrial content, gene expression (PGC1α, DRP1), metabolic activity (OXPHOS, β-oxidation) and biogenesis are immanent [77,78,90]. However, metabolic and respiratory activity might adapt after surviving critical illness, switching nutrient utilization of the remaining mitochondria to increased FFA oxidation. This phenomenon is seen up to 41 days after ICU admission [87,88]. These findings are underpinned by recent data showing divergent dynamics in overall mitochondrial integrity between survivors and non-survivors of critical illness, associating preservation and restoration of metabolic function with beneficial outcomes [194]. Observations regarding long-term changes to muscle mitochondrial structure and function are sparse. Six months after critical illness has been overcome, mitochondrial content may restore up to normal, but structural and possibly functional impairments might persist over months, accompanied by marked muscle weakness and wasting [83,195]. This is supported by recent data showing that even few days of critical illness may contribute to significant alterations of mitochondrial respiratory activity up to 6 months after ICU admission [196]. Considering the dynamics of mitochondrial dysregulation during the course of critical illness, several interesting attempts can be made to restore and improve mitochondrial function and patient outcome.

As insulin resistance and impaired glucose utilization have been linked to mitochondrial dysfunction promoting the development of neuromuscular failure in critically ill patients, glycemic control has become a promising treatment option. The use of an intensified insulin treatment has been shown to reduce the incidence of CINM and CIM [197,198]. The underlying molecular mechanisms of insulin directly contributing to improvements in mitochondrial function have yet to be fully explored, but may include increased mitochondrial respiratory capacity, gene expression and protein synthesis, as well as improved OXPHOS coupling efficacy [176,199,200]. Furthermore, intensified insulin treatment seems to increase GLUT4 mRNA expression in skeletal muscles, further supporting glycemic control [201]. In addition, non-pharmacological attempts to overcome insulin resistance and promote glucose utilization have been made, showing that electrical stimulation can help to translocate GLUT4 into the sarcolemmal membrane [84]. However, whether the direct action of insulin or the control of glycemic levels are accountable for the preservation and restoration of mitochondrial function may vary within different tissues, and remains debatable [202].

Beyond glucose metabolism, a differentiated and personalized nutritional regime might help to restore mitochondrial function in patients with critical illness, preventing ICUAW and CIM. The amino acids leucine and glutamine have been shown to promote activation of PGC1α and ETC complex I activity in skeletal muscles in a recent animal model [203]. The infusion of ketone bodies has been shown to protect from sepsis-induced muscle wasting [187]. Ketone bodies might help to fuel mitochondrial ATP production in catabolic conditions such as critical illness by improving oxidative metabolism [204]. However, the effect of a prolonged administration of ketone bodies on muscle function and integrity remains controversial.

Exercise might be an important non-pharmacological approach to improve mitochondrial function in critically ill patients. Early mobilization is safe, maintains muscle mass and prevents against ICUAW [205]. On the molecular level, muscle contractions activate the AMPK, which in turn promotes mitochondrial biogenesis, mitochondrial dynamics and mitophagy via activation of PGC1α [206]. Furthermore, the combination of exercise and insulin treatment might act synergistically and help to improve mitochondrial function in critical illness, possibly further decreasing the risk of ICUAW [207].

In order to reduce oxidative stress and counteract free oxygen radical damage, various antioxidative pharmacological agents have been evaluated to improve mitochondrial function and counteract muscle wasting and dysfunction. Thus, extensive research has been conducted on sepsis-induced diaphragm dysfunction using rodent animal models [208,209,210]. Treatment with various antioxidant drugs (e.g., MitoTEMPOL, SS31, mitoquinone mesylate) has been shown to reduce ROS concentrations, prevent myosin protein degradation and preserve contractile muscle force generation in diaphragm muscle strips. Regarding the molecular mechanism of action, it is believed that these molecules act directly on the IMM by scavenging ROS and, therefore, reducing mitochondrial oxidative stress. In this context, positive effects of antioxidant treatment have also been demonstrated for limb skeletal muscle function [211,212]. Unfortunately, to date, no studies evaluating these antioxidants in humans suffering from critical illness-induced muscle wasting are available.

Another approach to restore mitochondrial function in critical illness-induced muscle weakness is to promote mitochondrial biogenesis and to preserve the fusion–fission balance in mitochondrial dynamics. Pharmacologically-induced activation of NRF2 has been evaluated in an attempt to promote mitochondrial biogenesis and, therefore, to restore mitochondrial count, with positive results [213,214]. This seems conclusive, since a reduction in the mitochondrial content of the overall skeletal muscles is frequently observed in critical illness [101,102]. Beyond activation of biogenesis, inhibition of overshooting mitochondrial fission might also provide interesting attempts to overcome mitochondrial dysfunction. The mitochondrial fission inhibitor Mdivi-1 has been shown to prevent sepsis-induced organ dysfunction. Unfortunately, in this study, the effect on skeletal muscles was not evaluated.

## 8. Conclusions

In this narrative review, we illustrated the molecular patterns of mitochondrial function in skeletal muscle homeostasis, in both health and critical illness. Mitochondrial dysfunction seems to be involved in different aspects of neuromuscular failure in critical illness, contributing to critical illness-induced muscle weakness and, at least in part, to muscle wasting. Thus, impairment is present at different levels of mitochondrial structure and function. As mitochondria are involved in various cellular pathways, their failure leads to bio-energetic crises, which impair cell integrity. However, the majority of data on mitochondrial dysfunction related to neuromuscular failure in critical illness were derived from animal studies, whereas evidence from critically ill patients was sparse. Recent advances in diagnostics and monitoring of mitochondrial function deepened our understanding of the pathological mechanisms related to mitochondrial dysfunction in neuromuscular failure, although their value for daily clinical practice remains to be approved. Interesting therapeutic approaches have been made to preserve or restore mitochondrial metabolism and, therefore, muscle function, representing one important factor in the multiprofessional care of the critically ill. Future efforts should not only focus on the molecular patterns of skeletal muscle mitochondrial dysfunction in the acute phase, but also need to evaluate the adaptive processes in chronic states of critical illness.

## Data Availability

Not applicable.
